# Comparative Assessment of Corneal Imaging Devices for Pediatric Patients: Evaluating Keratometric Measurements and Wavefront Aberrations

**DOI:** 10.18502/jovr.v20.16152

**Published:** 2025-11-10

**Authors:** Renato Souza Oliveira, João Quadrado Gil, Maria João Quadrado, Mauro Campos

**Affiliations:** ^1^Instituto Brasileiro de Oftalmologia – IBOL, Rio de Janeiro, Brazil; ^2^Faculty of Medicine, University of Coimbra, Coimbra, Portugal; ^3^Clinical Academic Center of Coimbra, Coimbra, Portugal; ^4^Department of Ophthalmology and Visual Sciences, Paulista School of Medicine, Federal University of São Paulo – UNIFESP, São Paulo, Brazil; ^5^Centro de Responsabilidade Integrado de Oftalmologia, Centro Hospitalar e Universitário de Coimbra, Coimbra, Portugal

**Keywords:** Corneal Aberration, Corneal Tomography, Down Syndrome, Keratoconus; Pediatric Keratoconus

## Abstract

**Purpose:**

To evaluate the intrasession repeatability and agreement in keratometric and wavefront measurements among three different instruments (Pentacam HR, Nidek OPD-Scan III [OPD], and Zeiss i-Profiler ${}^{\text{Plus}}$ [IPROF]) in a pediatric population with various clinical features.

**Methods:**

This cross-sectional study included 217 eyes from 114 patients aged 6 to 17 years with different clinical features. The patients were divided into five groups: one control group (C) and four other groups, each presenting with keratoconus (KC), ocular allergy (OA), high astigmatism, or Down syndrome (DS). Statistical analyses included the intraclass correlation coefficient (ICC) for repeatability and Bland-Altman plots for agreement.

**Results:**

The findings demonstrated excellent repeatability of keratometric parameters across all devices (e.g., K1 ICC: 99.53% for Pentacam, 98.10% for OPD, and 98.31% for IPROF). The Pentacam showed superior repeatability for aberrometry indices in the KC group, with ICC values exceeding 98% for high-order aberration root mean square (HOA RMS) and Zernike polynomials. However, in the DS group, repeatability was significantly reduced for certain parameters, such as the index of surface variance (ICC: 40.13%) and HOA RMS (ICC: 42.86%). Bland-Altman plots revealed variations among devices in asphericity, vertical coma, and HOA RMS, with the KC group exhibiting broader limits of agreement compared to the control group.

**Conclusion:**

All three instruments showed good repeatability, with the Pentacam demonstrating superior reliability across all parameters, including aberrometry. However, agreement between devices was poor for key indices in patients with KC and DS.

##  INTRODUCTION

A myriad of imaging devices are used to assess the cornea and its properties, such as curvature, pachymetry, and aberrometry, which enable the diagnosis and monitoring of corneal diseases such as keratoconus (KC).^[1]^ Numerous publications support the reliability of these devices in adults.^[2,3,4]^ However, there is limited information in the literature regarding the repeatability and reproducibility of keratometric and wavefront measurements in both healthy and ectatic corneas of young individuals.

Pediatric corneal imaging poses unique challenges due to age-related factors, developmental stages, and varying tolerances to discomfort and/or light sensitivity. Additionally, many medical imaging devices rely on anatomical assumptions that may not be directly applicable to younger patients. These challenges are particularly critical for children with anatomical variations or clinical conditions where the risk of inaccurate or unreliable measurements is higher and the need for a precise and early diagnosis is paramount.^[5,6]^

In this context, the main goal of the present study was to evaluate the intrasession repeatability and agreement in keratometric and wavefront measurements achieved using three different instruments on pediatric patients with various clinical features.

##  METHODS 

The study received approval from the Research Ethics Committee of Federal University of S ao Paulo (CEP 1170/2020). All participants or their legal guardians signed informed consent forms tailored to their age and comprehension level.

### Participants and Procedures

This cross-sectional study adhered to the principles of the Declaration of Helsinki and was approved by the ethics committees of the participating institutions. Prior to the commencement of the study, all participants or their legal guardians signed informed consent forms tailored to their age and comprehension level.

Patients aged 6 to 17 years were evaluated using three devices: the Pentacam HR (Oculus, Wetzlar, Germany), the OPD Scanner III (Nidek Technologies, Gamagori, Japan), and the Zeiss i-Profiler ${}^{\text{Plus}}$ (Carl Zeiss Vision, Aalen, Germany). The same investigator conducted three consecutive measurements on each device on the same day using auto-tracking and auto-shot functions in accordance with the manufacturer's instructions. The order of the devices was randomized, with no specific starting device.

Testing was conducted on the patients' natural pupils under scotopic conditions. Patients were instructed to widen their eyelids as much as possible and focus on the internal fixation target. Scans were taken in the automatic release mode. Each participant had a brief rest period between the consecutive measurements to mitigate the effects of fatigue and ensure consistent focus.

The rest period was standardized across all participants, lasting approximately 2–3 minutes, which was considered sufficient to prevent significant changes in corneal hydration or structure.

By standardizing the time intervals and conditions during the measurement process, we aimed to ensure that the repeatability checks accurately reflect the performance of each device in a controlled setting.

Pentacam examinations were conducted in automatic mode, with a caption made in 2 seconds with the default setting of 25 images/second. OPD was set to 50% light intensity with normal backlighting, and IPROF was set to medium light intensity.

Quality assessment was performed across all devices (automatically on Pentacam and manually on OPD and IPROF), and only good-quality images were recorded for analysis. If the quality score was not acceptable, the measurement was repeated immediately, provided the patient was able to tolerate it.

Patients were categorized into five groups: keratoconus (KC), astigmatism > 2 diopters (D; Cyl), ocular allergy (OA), Down syndrome (DS), and control (C). The C group comprised patients who exhibited normal clinical examinations, met normal topographic criteria, and had < 1 spherical and 1 cylindrical diopter on refraction. We excluded participants with a history of ocular diseases (except OA), ocular surgeries, ocular trauma, corneal scarring, or contact lens use.

The investigated indices are listed below:

Internal anterior chamber depth (ACD), corneal volume (Cvol), flat (K1) and steep (K2) keratometric readings of the anterior and posterior cornea, mean curvature cornea keratometry within the central 3 mm circle (Km), maximum curvature cornea keratometry (Kmax), corneal asphericity (Q), corneal volume (Cvol), inferior-superior index (IS), index of surface variance (ISV), corneal thickness at the thinnest point (TP), Ambrosio's relational thickness maximum (ART-Max), Belin/Ambrosio Deviation Index (BAD-D), total aberration (TOA), low-order aberration (LOA), and high-order aberration (HOA) of the entire, anterior and posterior cornea expressed as root mean square (RMS) data, third-order Zernike polynomials of vertical coma (Z3, –1), third-order oblique trefoil (Z3, –3), and fourth-order spherical aberration, Z4,0 (SA).

All Zernike coefficients were calculated based on height data for a pupil diameter of 6.0 mm. Also, TOA, LOA, and HOA were expressed as RMS data.

We considered the following criteria in diagnosing KC: traditional slit-lamp KC signs (such as Vogt's striae), abnormal topographic parameters (a skewed asymmetric bow tie, central or inferior steepening, or a claw pattern on topography), Kmax > 47.2 D, and IS value > 1.4 D at 6 mm.^[7,8]^ To prevent misdiagnosis, two corneal specialists evaluated all examinations.

### Statistical Analysis

The data obtained from the devices were exported to a spreadsheet using Microsoft Excel 2007 software. The values used for statistical analyses were calculated from the average of the three consecutive measurements of each device.

A linear model with random effects was used to compare the mean indices and ages across groups, considering potential dependencies between the left and right eyes of the same patient. Descriptive analyses were performed using frequency tables.

Intra-device repeatability was assessed using the intraclass correlation coefficient (ICC), where a high ICC indicates highly similar and consistent observations.^[9,10]^

The ICC values and their confidence intervals (CI) were compared across the devices to determine which device provided the most consistent measurements.

Bland-Altman plots were used to visualize the measurement discrepancies and the agreement and interchangeability of measurements between devices.^[11]^

Statistical analyses were conducted using Stata/SE, 14.0 (Stata Corp, College Station, TX, USA), and *P*-values < 0.05 were considered statistically significant.

##  RESULTS

The study included 217 eyes from 114 patients, with a mean age of 12.78 ± 3.78 years (range, 7 to 17 years) and a male predominance of 60.5%. Patients were categorized into five groups: OA (15%), KC (21%), high astigmatism (37%), DS (14%), and C (13%). No statistically significant differences were found between these groups in terms of age or sex (*P* > 0.05).

### Repeatability of Each Device in Each Group

The ICC charts revealed excellent repeatability of the keratometric parameters across all three devices. In terms of aberrometry indices, the Pentacam demonstrated higher repeatability in the RMS measurements and Zernike polynomials compared to the OPD and IPROF [Table 1]. The CI for the measurements obtained via the Pentacam did not overlap with those from the OPD and IPROF, indicating a significant difference in repeatability between the devices. Notably, the OPD's ICC values were the lowest among the parameters.

**Table 1:** Repeatability of keratometric and wavefront measurements on three corneal imaging devices (ICC × 100 [95% CI])

**Parameter**	**PENTACAM**	**OPD**	**IPROF**
K1	99.53 (99.40 to 99.63)	98.10 (97.59 to 98.52)	98.31 (97.85 to 98.68)
K2	99.75 (99.68 to 99.80)	99.43 (99.28 to 99.56)	98.79 (98.47 to 99.06)
Cyl	98.46 (98.05 to 98.80)	93.47 (91.77 to 94.89)	98.55 (98.16 to 98.87)
Q	92.62 (90.75 to 94.18)	54.75 (46.71 to 62.35)	70.87 (64.78 to 76.33)
Total RMS	99.26 (99.07 to 99.43)	85.07 (81.47 to 88.16)	86.61 (83.35 to 89.39)
HOA RMS	98.02 (97.50 to 98.46)	39.10 (30.14 to 48.03)	61.48 (54.15 to 68.26)
Z(3,1)	97.97 (97.43 to 98.41)	51.24 (42.91 to 59.20)	95.86 (94.75 to 96.77)
Z(3,–1)	98.59 (98.22 to 98.90)	87.72 (84.68 to 90.30)	81.86 (77.59 to 85.54)
Z(3,–3)	74.67 (69.26 to 79.48)	12.13 (3.50 to 21.51)	51.97 (43.70 to 59.85)
Z(4,0)	99.13 (98.90 to 99.32)	70.02 (63.76 to 75.65)	82.61 (78.49 to 86.16)
	
	
CI, confidence interval; K1, flattest corneal meridian; K2, steepest corneal meridian; Cyl, topographic astigmatism; Q, asphericity at the central 6.0 mm zone; Total RMS, total root mean square value of the total corneal aberration; HOA, higher-order aberration RMS of the total cornea (from third- to sixth-order); Z(3,1), Zernike coefficient for primary horizontal coma; Z(3,–1), Zernike coefficient for primary vertical coma; Z(3,–3), vertical trefoil; Z(4,0), primary spherical aberration; OPD, Nidek OPD Scanner III; IPROF, Zeiss i-Profiler plus.			

**Table 2:** Repeatability of keratometric and wavefront measurements on Pentacam in each group (ICC × 100 [95% CI])

**Parameter**	**Ocular allergy**	**Keratoconus**	**Astigmatism**	**Down syndrome**	**Control**
K1	99.30	99.29	99.58	99.32	99.52
	(98.77 to 99.63)	(98.81 to 99.60)	(99.33 to 99.76)	(98.19 to 99.80)	(99.13 to 99.75)
K2	99.15	99.67	99.54	99.43	99.39
	(98.51 to 99.54)	(99.45 to 99.81)	(99.26 to 99.73)	(98.47 to 99.83)	(98.90 to 99.69)
Kmax	98.15	99.10	98.87	92.43	97.13
	(96.77 to 99.01)	(98.48 to 99.49)	(98.17 to 99.33)	(81.18 to 97.68)	(94.84 to 98.52)
TP	98.42	99.14	99.42	87.72	98.47
	(97.23 to 99.15)	(98.56 to 99.52)	(99.06 to 99.66)	(70.90 to 96.16)	(97.24 to 99.22)
ART MAX	85.58	96.49	76.67	74.86	81.76
	(76.35 to 91.94)	(94.17 to 98.00)	(65.41 to 85.36)	(47.17 to 91.59)	(69.76 to 90.10)
BAD-D	91.26	99.49	75.41	70.16	81.18
	(85.28 to 95.21)	(99.14 to 99.72)	(63.57 to 84.59)	(39.79 to 89.76)	(68.89 to 89.77)
ISV	59.18	99.72	98.87	40.13	90.97
	(40.47 to 75.03)	(99.52 to 99.84)	(98.17 to 99.33)	(3.70 to 75.42)	(84.30 to 95.25)
RMS total	75.84	99.23	98.17	88.64	81.78
	(62.11 to 86.05)	(98.71 to 99.57)	(97.02 to 98.94)	(72.83 to 96.46)	(69.79 to 90.11)
RMS HOA	68.89	99.40	88.40	42.86	52.57
	(52.70 to 81.61)	(98.98 to 99.66)	(81.83 to 93.05)	(6.36 to 76.95)	(31.36 to 71.36)
Z(3,–1)	91.47	99.13	91.14	58.24	85.43
	(85.61 to 95.32)	(98.54 to 99.51)	(86.10 to 94.67)	(23.48 to 84.67)	(75.38 to 92.19)
Z(4,0)	79.36	99.39	86.96	69.54	78.67
	(67.12 to 88.22)	(98.98 to 99.66)	(79.88 to 92.06)	(38.86 to 89.51)	(65.16 to 88.30)
CI, confidence interval; K1, flattest corneal meridian; K2, steepest corneal meridian; Kmax, maximum keratometry; TP, thinnest pachymetry point; ART MAX, maximum Ambrosio relational thickness; BAD-D, Belin/Ambrosio Deviation Index; ISV, index of surface variance; RMS total, total corneal aberration root mean square; RMS HOA, high-order corneal aberration root mean square; Z(3,–1), Zernike coefficient for primary vertical coma; Z(4,0), primary spherical aberration.					

**Table 3:** Multilevel mixed models for each of the three corneal imaging devices

	**Mean ± SD**	**Coefficient (95%CI)**	***P*** **-value**

K1			
PENTACAM	43.38 D ± 2.40	Reference	—
OPD	43.49 D ± 2.23	0.17 (–0.03 to 0.37)	0.089
IPROF	43.51 D ± 2.22	0.17 (–0.02 to 0.37)	0.086
K2			
PENTACAM	46.02 D ± 3.11	Reference	—
OPD	46.19 D ± 3.21	0.22 (–0.09 to 0.53)	0.158
IPROF	46.27 D ± 3.04	0.28 (–0.02 to 0.59)	0.071
Km			
PENTACAM	44.64 D ± 2.61	Reference	—
OPD	45.08 D ± 4.74	0.54 (0.02 to 1.06)	0.043
Cyl			
PENTACAM	2.64 D ± 1.73	Reference	—
OPD	2.68 D ± 1.94	0.03 (–0.12 to 0.18)	0.702
IPROF	2.76 D ± 1.84	0.12 (–0.03 to 0.27)	0.129
Q			
PENTACAM	–0.52 ± 0.26	Reference	—
OPD	–0.70 ± 6.23	–0.18 (–0.86 to 0.50)	0.603
IPROF	–0.47 ± 0.17	0.05 (–0.63 to 0.72)	0.893
TOTAL RMS			
PENTACAM	3.49 ± 3.03	Reference	—
OPD	4.36 ± 3.97	0.87 (0.50 to 1.23)	< 0.001
IPROF	3.19 ± 2.06	–0.30 (–0.67 to 0.06)	0.102
HOA RMS			
PENTACAM	0.71 ± 0.79	Reference	—
OPD	1.08 ± 1.53	0.37 (0.20 to 0.53)	< 0.001
IPROF	0.93 ± 1.12	0.22 (0.05 to 0.38)	0.009
Z(3,–1)			
PENTACAM	–0.24 μ m ± 0.65	Reference	—
OPD	–0.27 μ m ± 0.76	–0.05 (–0.13 to 0.03)	0.226
IPROF	–0.26 μ m ± 0.87	–0.02 (–0.10 to 0.06)	0.558
Z(3,–3)			
PENTACAM	0.02 μ m ± 0.30	Reference	—
OPD	0.07 μ m ± 1.44	0.05 (–0.11 to 0.22)	0.513
IPROF	0.12 μ m ± 0.47	0.10 (–0.06 to 0.26)	0.205
Z(4,0)			
PENTACAM	0.05 μ m ± 0.40	Reference	—
OPD	0.00 μ m ± 0.39	–0.06 (–0.11 to –0.01)	0.025
IPROF	0.05 μ m ± 0.49	0.00 (–0.05 to 0.05)	0.980
SD, standard deviation; CI, confidence interval; K1, flattest corneal meridian; K2, steepest corneal meridian; Cyl, topographic astigmatism; Q, asphericity at the central 6.0 mm zone; Total RMS, total corneal aberration R HOA RMS, high-order corneal aberration R Z(3,–1), Zernike coefficient for primary vertical coma; Z(3,–3), vertical trefoil; Z(4,0), primary spherical aberration.			

**Figure 1:**
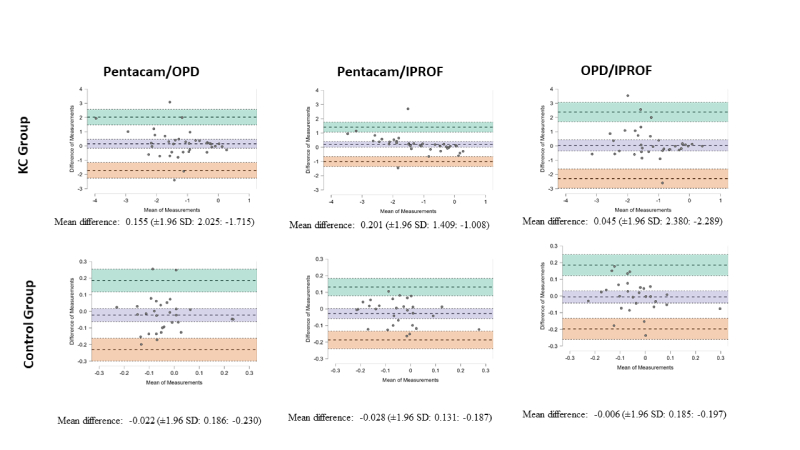
Bland-Altman plots for vertical coma in the KC and control groups.

**Figure 2:**
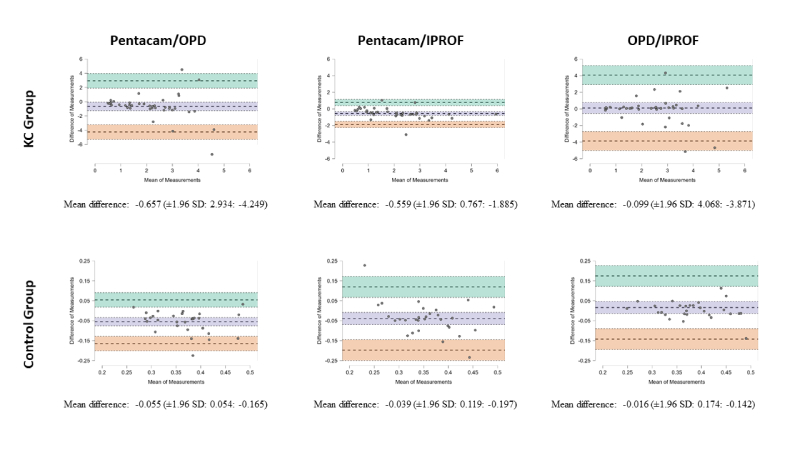
Bland-Altman plots for HOA RMS in the KC and control groups.

When evaluating repeatability within each group independently, we conducted analyses on 29 indices using the Pentacam, 11 with the OPD, and 11 with the IPROF. In general, the Pentacam demonstrated superior ICC values, indicating excellent repeatability for nearly every parameter within the KC group. However, within the DS group, repeatability was less favorable for specific parameters, particularly ISV and aberrometry-derived indices. The OPD and IPROF devices displayed a similar trend, although their repeatability was not as good as that observed with the Pentacam. The OA, Cyl, and C groups demonstrated good to excellent ICC values across most indices, with only a few clinically insignificant exceptions.

Table 2 displays ICC values for selected analyzed indices on the Pentacam. Indices designed to identify or predict the risk of KC development, such as BAD-D, maximum Ambrosio relational thickness (ART-max), and ISV, exhibited good or excellent values in almost every group. The comprehensive Pentacam analyses, as well as those from the OPD and IPROF, are available in the Supplementary Materials [Supplementary Tables 1–3].

### Comparison of Measurements Between the Devices

Most of the analyzed indices did not exhibit statistically significant differences among the three devices. However, the OPD tended to yield higher values for both total aberration and HOA RMS than the Pentacam [Table 3].

An analysis of Bland-Altman plots for vertical coma and HOA RMS revealed that in both the KC and C groups, OPD and IPROF exhibited a lower mean difference when compared to measurements obtained with the Pentacam. However, they also exhibited wider limits of agreement [Figures 1 & 2].

In the KC group, the mean differences (biases) for vertical coma and HOA RMS were smaller between the OPD and IPROF (0.045 and 0.099) compared to those between the Pentacam and OPD (0.155 and 0.657) or between the Pentacam and IPROF (0.201 and 0.559). In the C group, the mean differences for vertical coma and HOA RMS were also smaller between OPD and IPROF (0.006 and 0.016) compared to those between Pentacam and OPD (0.022 and 0.055) or between Pentacam and IPROF (0.028 and 0.039).

Similarly, in the C group, the biases were at most 0.028 for the vertical coma and 0.055 for HOA RMS. Furthermore, the limits of agreement for all comparisons, including those involving Pentacam, were significantly lower in the C group compared to the KC group.

##  DISCUSSION

Numerous devices provide corneal measurements based on distinct optical principles and specific algorithms. However, these measurements are often treated as if they were interchangeable. This incongruity is particularly noticeable in pediatric populations, especially in those with distinct clinical or anatomical attributes. Furthermore, these measurements must demonstrate reliability within specific age groups. In our study, although each of the three devices exhibited consistent internal repeatability, significant trends in variation were observed among them.

The Oculus Pentacam HR uses a 475 nm blue LED light and a Scheimpflug rotating camera, capturing images at 25 frames/second. Its software converts corneal elevation profiles into wavefront data using Zernike polynomials up to the 10 ${}^{\text{th}}$ order.^[2,12]^ The Nidek OPD-Scan III combines a Hartmann–Shack aberrometer and a Placido-based topographer, measuring aberrations up to the 8 ${}^{\text{th}}$ order.^[4,13]^ The Zeiss i-Profiler Plus uses an infrared laser and HS microlens-array sensor, quantifying wavefronts up to the 7 ${}^{\text{th}}$ radial order.^[14,15]^ These differences in basic principles could account for the observed variations in outcomes. These variations are further influenced by the duration of image acquisition and the light intensity used by each device.

While all three devices exhibited excellent repeatability for keratometric parameters, only the Pentacam showed favorable values for asphericity and aberrometry indices. Furthermore, it demonstrated outstanding repeatability for pachymetric-derived indices, which is consistent with previous studies.^[2,3,16,17]^ However, the repeatability of these devices tends to decrease in patients with KC. Several authors have highlighted that KC negatively affects the repeatability of corneal parameters across various devices.^[4,18]^ Similarly, Hashemi et al observed that corneal tomography repeatability decreases with increasing KC severity,^[19]^ and Kreps et al reported lower repeatability in moderate KC compared to C or mild disease groups, especially for BAD-D and Kmax.^[20]^

A recent study by Sena et al noted that adult patients with KC showed greater variation in repeatability across all indices with the Pentacam compared to those with normal eyes.^[21]^ In our study, however, all devices demonstrated excellent repeatability across various parameters in children with KC. Most aberrometry values showed excellent repeatability for the KC group across all devices. This result aligns with studies reporting high precision for anterior and posterior corneal wavefront aberrations in normal and keratoconic eyes using the Pentacam,^[22]^ OPD,^[4]^ and IPROF.^[14]^

Although few studies have evaluated the IPROF's repeatability, Liao et al demonstrated its ability to provide highly repeatable high-order aberration measurements in healthy individuals.^[14]^ In our study, the repeatability of IPROF was consistent with previous findings, but its agreement with other devices remains limited. To our knowledge, this is the first study assessing the agreement between Zeiss IPROF and other corneal imaging devices for keratometry and aberrometry measurements.

Shetty et al found that Pentacam, Galilei, and Sirius, although based on the same principle—the Scheimpflug technology—cannot be used interchangeably for anterior segment measurements in patients with KC.^[23]^ Similarly, in our study, notable differences were observed among the outputs of Pentacam, OPD, and IPROF. Bland-Altman analysis revealed that while RMS HOA measurements in the C group could be considered interchangeable across devices, this was not the case for the KC group, where mean variations were 0.66 µm between Pentacam and OPD and 0.56 µm between Pentacam and IPROF. A similar pattern was seen for vertical coma. Despite smaller mean differences between OPD and IPROF, their limits of agreement were considerably wider, suggesting these devices are no more interchangeable than with the Pentacam.

We chose to conduct an in-depth study of selected indices, such as vertical coma and HOA RMS, because the literature has proven that they are helpful in detecting established or forme fruste KC.^[24,25]^

This study is not without limitations. Dynamic changes in the tear film and subtle eye movements could have influenced measurements, particularly in children, whose fixation may deviate from optimal.^[2,26]^ These factors underscore the importance of patience, clear instructions, and repeated attempts during pediatric imaging. Additionally, while we examined asphericity over a 6.0 mm area, prior studies suggest better repeatability within a 7.0 mm zone.^[16]^ Future studies could explore this further. Finally, we did not stratify patients with KC by severity, which could have influenced our findings, as severity significantly impacts repeatability and agreement.^[20,21]^

Our findings highlight that as corneal irregularity increases, agreement among devices diminishes. Ophthalmologists should exercise caution when interpreting results from different devices, particularly for pediatric patients or those with ocular abnormalities. Data from this study provide evidence-based reassurance for clinicians navigating these challenges in real-world practice.

In summary, the Pentacam demonstrated good intrasession repeatability in a pediatric cohort and surpassed the performance of both OPD and IPROF, even among patients with KC, high astigmatism, or DS. Additionally, inter-device agreement is not favorable for specific and critical indices such as aberrometry values in patients with KC.

##  Financial Support and Sponsorship

None.

##  Conflicts of Interest

None.
